# Updated birth prevalence and relative frequency of mucopolysaccharidoses across Brazilian regions

**DOI:** 10.1590/1678-4685-GMB-2020-0138

**Published:** 2021-01-27

**Authors:** Juliana Alves Josahkian, Franciele Barbosa Trapp, Maira Graeff Burin, Kristiane Michelin-Tirelli, Ana Paula Pereira Scholz de Magalhães, Fernanda Medeiros Sebastião, Fernanda Bender, Jurema Fátima De Mari, Ana Carolina Brusius-Facchin, Sandra Leistner-Segal, Diana Rojas Málaga, Roberto Giugliani

**Affiliations:** 1 Hospital Universitário de Santa Maria, Unidade de Clínica Médica, Santa Maria, RS, Brazil.; 2 Universidade Federal do Rio Grande do Sul, Programa de Pós-Graduação em Genética e Biologia Molecular, Porto Alegre, RS, Brazil.; 3 Rede MPS Brasil, Porto Alegre, RS, Brazil.; 4 Hospital de Clínicas de Porto Alegre, Serviço de Genética Médica, Porto Alegre, RS, Brazil.; 5 Hospital de Clínicas de Porto Alegre, Centro de Pesquisa Experimental, Grupo de Pesquisa BIODISCOVERY, Porto Alegre, RS, Brazil.; 6 Grupo Fleury, Pesquisa e Desenvolvimento - Biologia Molecular, São Paulo, SP, Brazil.; 7 Universidade Federal do Rio Grande do Sul, Departamento de Genética, Porto Alegre, RS, Brazil.; 8 Instituto Nacional de Genética Médica Populacional (INAGEMP), Porto Alegre, RS, Brazil.; 9 Hospital de Clínicas de Porto Alegre, Centro de Pesquisa Clínica, Grupo de Pesquisa DRBRASIL, Porto Alegre, RS, Brazil.

**Keywords:** Lysosomal storage diseases, metabolic diseases, mucopolysaccharidoses, epidemiology, Brazil

## Abstract

The mucopolysaccharidoses (MPS) are a group of lysosomal storage disorders caused by 11 enzyme deficiencies, classified into seven types. Data on the birth prevalence of each MPS type are available for only a few countries, and the totality of cases may be underestimated. To determine the epidemiological profile of MPS in each Brazilian region, we analyzed data collected between 1982 and 2019 by a national reference laboratory and identified 1,652 patients. Using data between 1994 and 2018, the birth prevalence (by 100,000 live births) for MPS was 1.57. MPS II was the most common type of MPS in Brazil, and its birth prevalence was 0.48 (0.94 considering only male births). Regarding the number of cases per region, MPS II was the most frequent in the North and Center-West (followed by MPS VI), and also in the Southeast (followed by MPS I); MPS I and MPS II were the most common types in the South; and MPS VI was the most common in the Northeast (followed by MPS II). The differences observed in the relative frequencies of MPS types across Brazilian regions are likely linked to founder effect, endogamy, and consanguinity, but other factors may be present and need further investigation.

## Introduction

Mucopolysaccharidoses (MPS) are a group of lysosomal storage disorders caused by the deficiency of enzymes involved in the catabolism of glycosaminoglycans (GAGs). These conditions are multisystemic, progressive, and have variable clinical features (Neufeld and Muenzer, 2014), not only among the different types but also among patients with the same type of MPS. Severe cases are easier to diagnose, but attenuated cases are challenging to recognize and can be confounded with more common pathologies (Suarez-Guerrero *et al.*, 2015).

Studies of the specific enzymes involved in different steps of the GAG degradation pathway and the identification of which genes cause the disease allowed the classification of MPS in seven clinical types, which correspond to 11 enzyme deficiencies, currently recognized as MPS I, II, III (A, B, C, and D subtypes), IV (A and B subtypes), VI, VII, and IX. All MPS are autosomal recessive disorders, except MPS II, which is an X-linked recessive condition (Neufeld and Muenzer, 2014).

Epidemiological data about the MPS types are available for only a few countries and regions, and its birth prevalence may be underestimated as a consequence of the clinical heterogeneity of this group of diseases and the difficulties for its laboratory investigation (Giugliani, 2012). For this reason, a laboratory to provide diagnostic support for MPS was established at the Medical Genetics Service of Hospital de Clínicas de Porto Alegre (MGS/HCPA), Brazil. MGS/HCPA is a well-known reference center in the country, and it has received samples from patients with suspected MPS since 1982 (Giugliani *et al.*, 2017). In 2004, the demand for testing patients suspected of having MPS led to the creation of the MPS Brazil Network, with a specific investigation workflow (Giugliani *et al.*, 2016). In this manner, this study aimed to report the birth prevalence and relative frequency of the different MPS types in Brazil to determine the epidemiological profile of this condition per state, per region and in the country as a whole.

## Material and Methods

We analyzed the records from the MGS/HCPA and the MPS Brazil Network of patients diagnosed with MPS between 1982 and 2019. MPS cases were diagnosed biochemically; the investigation frequently starts with a quantitative (colorimetric method with dimethylene blue) and qualitative (electrophoresis) analysis of urinary GAGs, followed by specific enzyme assays according to the first results and/or by identification of pathogenic variants by molecular genetic analysis. In this study, we calculated the relative frequency of each MPS type in Brazil, and also present data by region and state. 

In this report we use the term *birth prevalence* to refer to the number of MPS cases diagnosed by the total number of live births in a specific period expressed as cases per 100,000 live births, as employed previously in the literature (Poupetová *et al.*, 2010; Khan *et al.*, 2017).

Data regarding live births from the Brazilian Health System database were available from 1994 to 2018, allowing us to calculate the birth prevalence in this period. Patients with MPS who were not born during this period were not included in the analyzes. The comparison between our findings and the estimations from other countries is also presented. 

## Results

From 1982 to 2019, 1,652 Brazilian patients were diagnosed with MPS at the Medical Genetics Service of Hospital de Clínicas de Porto Alegre. MPS II was the most commonly diagnosed condition (493 cases, 29.84%), followed by MPS VI (351 cases, 21.25%), MPS I (315 cases, 19.07%), MPS III - all subtypes (267 cases, 16.16%), MPS IV - both subtypes (205 cases, 12.41%) and MPS VII (21 cases, 1.27%). We did not observe any patients diagnosed with MPS IX.

Regarding MPS III, we identified the specific subtype (A, B, C, or D) for 95.50% of the cases. In this subset, the proportion of MPS IIIA was 26.67%; for IIIB, 45.49%; for IIIC, 27.45%; and there was just one case of IIID (0.39%). 

The same approach was employed for MPS IV. In 96.09% of the cases, we were able to identify each specific subtype (A or B). In this subset, the proportion of MPS IVA was 96.45%, and the percentage of MPS IVB was 3.55%. By extrapolating these data to the total number of MPS III and MPS IV cases, we calculated the ratios presented in Table 1.

**Table 1 -:** MPS types diagnosed in Brazil per region and by state^a^ from 1982 to 2019.

Region/State	MPS Type	All	I	II	IIIA	IIIB	IIIC	IIID	IVA	IVB	VI	VII	IX
North		75	13	30	2	4	2	-	5	-	18	1	-
Acre		7	2	1	-	1	-	-	2	-	1	-	-
Amazonas		31	6	10	1	1	-	-	1	-	12	-	-
Amapá		-	-	-	-	-	-	-	-	-	-	-	-
Pará		28	3	13	1	2	2	-	1	-	5	1	-
Rondônia		7	1	5	-	-	-	-	1	-	-	-	-
Roraima		1	-	1	-	-	-	-	-	-	-	-	-
Tocantins		1	1	-	-	-	-	-	-	-	-	-	-
Center-West		103	20	33	6	8	3	-	6	-	24	3	-
Distrito Federal		49	12	9	4	4	2	-	4	-	13	1	-
Goiás		22	2	11	2	-	-	-	1	-	6	-	-
Mato Grosso		15	5	6	-	3	-	-	1	-	-	-	-
Mato Grosso do Sul		17	1	7	-	1	1	-	-	-	5	2	-
Southeast		679	140	207	33	56	37	-	69	6	124	7	-
Espírito Santo		22	-	11	1	4	2	-	-	-	3	1	-
Minas Gerais		135	33	22	7	15	5	-	15	-	36	2	-
São Paulo		388	86	126	21	23	24	-	36	6	62	4	-
Rio de Janeiro		134	21	48	4	14	6	-	18	-	23	-	-
Northeast		510	66	147	22	14	19	1	68	1	162	10	-
Alagoas		34	4	23	1	-	1	-	-	-	5	-	-
Bahia		111	15	34	6	7	1	1	9	1	31	6	-
Ceará		88	11	35	5	6	3	-	7	-	21	-	-
Maranhão		23	2	11	2	1	-	-	3	-	4	-	-
Paraíba		71	8	6	1	-	9	-	26	-	20	1	-
Pernambuco		118	15	20	2	-	5	-	17	-	58	1	-
Piauí		19	2	7	1	-	-	-	2	-	5	2	-
Rio Grande do Norte		31	6	4	2	-	-	-	4	-	15	-	-
Sergipe		15	3	7	2	-	-	-	-	-	3	-	-
South		285	76	76	8	40	12	-	50	-	23	-	-
Paraná		102	20	39	3	12	3	-	13	-	12	-	-
Rio Grande do Sul		142	42	32	3	21	6	-	28	-	10	-	-
Santa Catarina		41	14	5	2	7	3	-	9	-	1	-	-
Brazil, total		1652	315	493	71	122	73	1	198	7	351	21	-

^a^ For MPS IIIA, IIIB, IIIC, and IIID, and for MPS IVA and IVB, the numbers represent an extrapolation.

**Figure 1 -:**
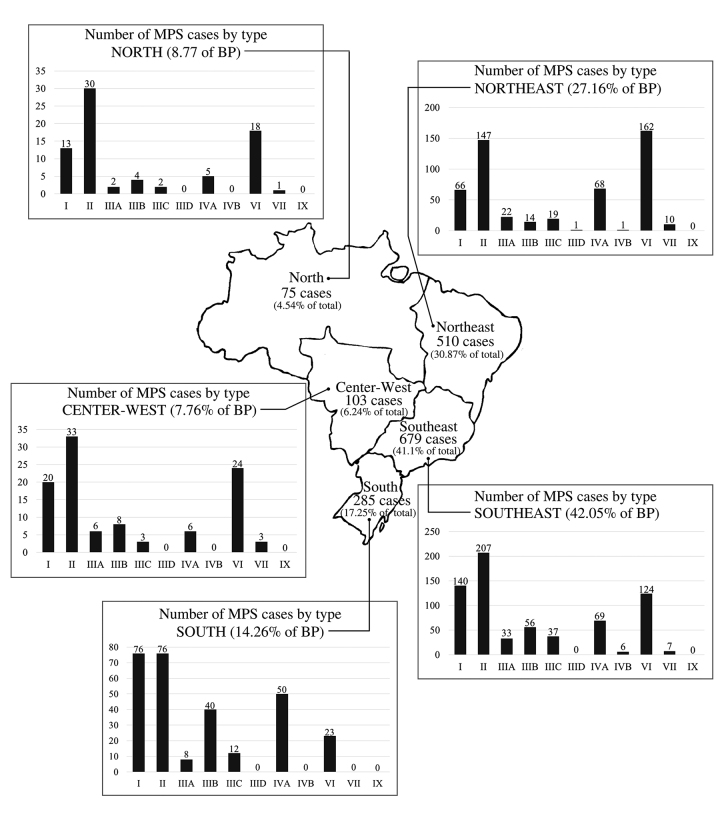
Distribution according to Brazilian region of the Brazilian MPS cases diagnosed at the Medical Genetics Service of Hospital de Clínicas de Porto Alegre from 1982 to 2019 (BP, Brazilian population as estimated in 2019) (IBGE, 2020).

When considering the number of cases diagnosed from each Brazilian region, we found that MPS II was the most frequent in the North, Center-West, and Southeast regions; MPS I and MPS II were tied as the most common types in the South region; and MPS VI was the most frequent in the Northeast region. The number of cases diagnosed according to the Brazilian region and state of origin is shown in Table 1, and the distribution of these types of MPS in Brazil is shown in Figure 1.

Based on data provided by the Information System on Live Births (SINASC) of the Brazilian Health System database, between 1994 and 2018, a total of 74,215,086 live births occurred in Brazil - 37,977,308 being male babies (DATASUS, 2020). We are aware of 1,164 Brazilian patients diagnosed with MPS who were born in Brazil during this period. Among these patients, 217 were MPS I, 358 were MPS II, 199 were MPS III (54 IIIA, 84 IIIB, 50 IIIC, 1 IIID, and 10 were MPS III not specified), 117 were MPS IV (110 MPS IVA, 2 MPS IVB, and five with MPS IV not specified), 257 were MPS VI and 16 were MPS VII. For calculation purposes, the unspecified cases were distributed proportionally according to the frequency of MPS subtype. Thus, the numbers were adjusted to 57 for MPS IIIA, 88 for MPS IIIB, 53 for MPS IIIC, and 115 for MPS IVA. 

The calculated incidence for MPS in Brazil, using the 1994 to 2018 data, was 1.57/100,000 live births. Regarding each MPS type, the birth prevalence by 100,000 live births was 0.29 for MPS I, 0.48 for MPS II (or 0.94, considering only male births), 0.08 for MPS IIIA, 0.12 for MPS IIIB, 0.07 for MPS IIIC, 0.001 for MPS IIID, 0.15 for MPS IVA, 0.003 for MPS IVB, 0.35 for MPS VI, 0.02 for MPS VII, and 0 for MPS IX. 

The birth prevalence was also calculated for each Brazilian region. For the 358 patients (30.76%) without an informed place of birth, the region from where samples were obtained was set as “place of birth.” Our results showed that MPS II had the highest score in all Brazilian regions, except in the Northeast, where MPS VI presented the highest birth prevalence rate. The number of cases diagnosed and the birth prevalence for MPS patients born from 1994 to 2018 in Brazil and each region of this country is detailed in Table 2.

**Table 2 -:** Number of cases diagnosed and incidence (by 100,000 live births)^a^ calculated for MPS patients born from 1994 to 2018 in Brazil and by region.

Region	MPS	All	I	II	IIIA	IIIB	IIIC	IIID	IVA	IVB	VI	VII	IX
North		65 (0.88)	13 (0.18)	28 (0.38) (0.74)^b^	2 (0.03)	4 (0.05)	2 (0.03)	-	4 (0.05)	-	11 (0.15)	1 (0.01)	-
Northeast		379 (1.78)	52 (0.25)	109 (0.51) (1.00)^b^	18 (0.08)	13 (0.06)	14 (0.07)	1 (0.005)	40 (0.19)	-	123 (0.58)	9 (0.04)	-
Center-West		73 (1.26)	14 (0.24)	27 (0.47) (0.91)^b^	2 (0.03)	8 (0.14)	1 (0.02)	-	2 (0.03)	-	17 (0.29)	2 (0.03)	-
Southeast		476 (1.62)	98 (0.33)	143 (0.49) (0.95)^b^	27 (0.09)	41 (0.14)	27 (0.09)	-	43 (0.15)	2 (0.007)	91 (0.31)	4 (0.01)	-
South		171 (1.66)	40 (0.39)	51 (0.49) (0.97)^b^	8 (0.08)	22 (0.21)	9 (0.09)	-	26 (0.25)	-	15 (0.15)	-	-
Brazil, total		1164 (1.57)	217 (0.29)	358 (0.48) (0.94)^b^	57 (0.08)	88 (0.12)	53 (0.07)	1 (0.001)	115 (0.15)	2 (0.003)	257 (0.35)	16 (0.02)	-

^a^ The incidence rate (shown inside parenthesis) was calculated using our data and the number of live births obtained from SINASC; ^b^ Considering only male live births.

This study was approved by the Ethics Committee of Universidade Federal do Rio Grande do Sul, Brazil (CAAE #82189417.5.0000.5347). This study was conducted in accordance with the ethical standards from the 1964 Declaration of Helsinki and its later amendments. Our manuscript does not contain data from any individual person.

## Discussion

In this study, we explored the epidemiological data of MPS in Brazil. MPS II was the most common type of MPS in Brazil and the second most common lysosomal storage disease diagnosed in our laboratory in previously published studies conducted by our group (Giugliani *et al.*, 2017). Since Brazil has continental dimensions, analysis per region was critical for showing that MPS II is the most common in the North, Southeast, and Center-West. Indeed, MPS I and MPS II were tied as the most common types in the South, and MPS VI was the most frequent in the Northeast.

Birth prevalence rates calculated from 1994 to 2018 indicated that MPS II was the most frequent in all regions except the Northeast, where MPS VI has the highest rate. A founder effect that resulted in a high frequency of p.H178L pathogenic variant in the *ARSB* gene, responsible for MPS VI, may explain the high number of cases in Brazil’s Northeast (Federhen *et al.*, 2020). This region also has areas of geographical isolation, endogamy, and a high number of consanguineous marriages that may lead to increased rates of MPS VI patients (Costa-Motta *et al.*, 2014; Vairo *et al.*, 2015). In addition, the birth prevalence of MPS II in the Northeast was similar to the one observed in other regions of Brazil, which suggests that the higher incidence of MPS VI is related to these factors, and not to a lower absolute number of births with MPS II. Similarly, as in Brazil, MPS II is the most common type found in Estonia, Taiwan, Japan, South Korea, China, and Switzerland (Krabbi *et al.*, 2012; Cho *et al.*, 2014; Chen *et al.*, 2016; Khan *et al.*, 2017). The higher birth prevalence of MPS II in East Asia was suggested to be a consequence of the p.R468 pathogenic variants in the *IDS* gene (Khan *et al.*, 2017). In South Korea, *IDS-IDS2* recombination mutations were the most frequently (Cho *et al.*, 2014). Molecular analysis of 103 unrelated South-Americans (including 91 Brazilian individuals) MPS II patients showed that small insertions, deletions, indels, and point mutations in the *IDS* gene were responsible for the disease in 81% of cases. Inversion/disruption or partial/total deletions of the *IDS* gene were found in 19% of the patients, and only eight pathogenic variants were found in more than one unrelated patient (Brusius-Facchin *et al.*, 2014). We do not have information about the rate of “de novo” mutation in the *IDS* gene in Brazil, but data from Latin America the literature estimate it as 10% (Amartino *et al.*, 2014).

A limitation of our study is that, although responsible for the vast majority of MPS diagnosis in Brazil, our laboratory is not the only to perform such tests, and some Brazilian cases may have been not included. Also, milder cases that are challenging to diagnose may be overlooked. Another estimate of the birth prevalence of MPS in Brazil, based on the frequency of heterozygotes for the most common pathogenic variant of the *IDUA* gene (p.Trp402Ter) in healthy blood donors and on the relative frequency of homozygosity for such variant in MPS I patients (Federhen *et al.*, 2020) was reported as 4.62/100,000 live births, nearly three times higher than the one found in this study (1.57). In this manner, although providing a comprehensive picture of the epidemiological profile of MPS in Brazil, the absolute numbers found in this study are possibly underestimated. A newborn screening (NBS) program would be more accurate to estimate the incidence of MPS. Although MPS testing is not included in the public NBS program in Brazil, pilot studies are being carried out in order to evaluate its feasibility for future incorporation (Camargo Neto *et al.*, 2018).

A previous estimation of the birth prevalence of MPS in Brazil was published with data from 1994 to 2015 (Federhen *et al.*, 2020). We have updated the birth prevalence across Brazilian regions up to 2018 and also demonstrated the distribution of MPS across each Brazilian State. We think this revision is important since the inclusion of only three years already demonstrated a change in the estimated birth prevalence of MPS by type in the South Region, where MPS I was previously the most common (Federhen *et al.*, 2020). Moreover, the knowledge of the distribution by state, which does not necessarily reflects the distribution by region, can help the design of targeted public policies. This report provides a comprehensive characterization of the epidemiological profile of the different MPS subtypes in Brazil and its variations across states and regions. The birth prevalence of MPS is variable across countries and regions and is likely linked to founder effect, endogamy, and consanguinity, but other factors that are still unclear may be present and may need further investigation. Our findings may help the assess of health needs in distinct populations and the delivery of medical care for these rare diseases.
